# Furaquinocins K and L: Novel Naphthoquinone-Based Meroterpenoids from *Streptomyces* sp. Je 1-369

**DOI:** 10.3390/antibiotics11111587

**Published:** 2022-11-10

**Authors:** Stepan Tistechok, Marc Stierhof, Maksym Myronovskyi, Josef Zapp, Oleksandr Gromyko, Andriy Luzhetskyy

**Affiliations:** 1 Department of Genetics and Biotechnology, Ivan Franko National University of Lviv, 79005 Lviv, Ukraine; 2 Department of Pharmaceutical Biotechnology, Saarland University, 66123 Saarbruecken, Germany; 3 Department of Pharmaceutical Biology, Saarland University, 66123 Saarbruecken, Germany; 4 Microbial Culture Collection of Antibiotic Producers, Ivan Franko National University of Lviv, 79005 Lviv, Ukraine; 5 Helmholtz Institute for Pharmaceutical Research Saarland, 66123 Saarbruecken, Germany

**Keywords:** furaquinocins, streptomycetes, acetylhydrazone, biological activity, meroterpenoids, rhizosphere microorganisms

## Abstract

Actinomycetes are the most prominent group of microorganisms that produce biologically active compounds. Among them, special attention is focused on bacteria in the genus *Streptomyces*. Streptomycetes are an important source of biologically active natural compounds that could be considered therapeutic agents. In this study, we described the identification, purification, and structure elucidation of two new naphthoquinone-based meroterpenoids, furaquinocins K and L, from *Streptomyces* sp. Je 1-369 strain, which was isolated from the rhizosphere soil of *Juniperus excelsa* (Bieb.). The main difference between furaquinocins K and L and the described furaquinocins was a modification in the polyketide naphthoquinone skeleton. In addition, the structure of furaquinocin L contained an acetylhydrazone fragment, which is quite rare for natural compounds. We also identified a furaquinocin biosynthetic gene cluster in the Je 1-369 strain, which showed similarity (60%) with the furaquinocin B biosynthetic gene cluster from *Streptomyces* sp. KO-3988. Furaquinocin L showed activity against Gram-positive bacteria without cytotoxic effects.

## 1. Introduction

Actinomycetes are aerobic, spore-forming, Gram-positive bacteria that are characterized by a high G+C content in their genomes. These bacteria synthesize approximately two-thirds of all natural antibiotics used in medicine, veterinary medicine, and agriculture [1]. On average, actinomycete strains have the genetic potential to produce approximately 10–20 secondary metabolites [2]. Secondary metabolites produced by actinomycetes have been widely studied since the 1950s and are the most economically and biotechnologically valuable source for discovering new biologically active compounds [3]. The analysis of actinomycete secondary metabolites resulted in the discovery of many biologically active compounds, which were later embodied in the development of antimicrobial (i.e., vancomycin, chloramphenicol, and tetracycline) anticancer (i.e., daunorubicin and bleomycin), and immunosuppressive (i.e., rapamycin) drugs among others [4]. However, due to the rapid spread of microbial pathogens and their frequent resistance to known antibiotics, the search for new biologically active compounds is still relevant.

Meroterpenoids are a large class of natural products derived from polyketide or nonpolyketide and terpenoid biosynthesis [5]. A significant amount of naphthoquinone-based meroterpenoids have been isolated from marine and soil-derived streptomycete bacteria [6,7]. The structure of naphthoquinone-based meroterpenoids contains a 1,3,6,8-tetrahydroxynaphthalene core and a prenyl moiety, which are linked by aromatic prenyltransferase enzymes [8]. To form the final structure, the resulting hybrid compound is then subjected to several cyclization and modification steps. These compounds exhibit an impressive spectrum of biological activities, namely, antitumour, antibacterial, antifungal, antimalarial, cytotoxic, anti-HIV, and neuroprotective activities, among others [9].

Our research focused on the discovery of new biologically active metabolites from soil-derived actinomycetes. *Streptomyces* sp. strain Je 1-369 was isolated from *Juniperus excelsa* (Bieb.) rhizosphere soil collected in the Crimean Peninsula (Ukraine). Previous studies of actinomycetes from this area led to the identification of several new compounds, viz., kendomycin E [10], albaflavenol B [11], juniperolide A [12], leopolic acid A [13], and others. In this study, we described the isolation, purification, and structure elucidation of two new naphthoquinone-based meroterpenoids, furaquinocins K and L, from *Streptomyces* sp. Je 1-369, which expands the structural diversity of the furaquinocin family of natural products. In addition, the furaquinocin biosynthetic gene cluster in the genome of the Je 1-369 strain was identified. The new furaquinocin L showed potent activity against Gram-positive bacteria without cytotoxic effects.

## 2. Results

### 2.1. Identification and Structure Elucidation of Furaquinocins K and L

The *Streptomyces* sp. Je 1-369 strain was isolated from the rhizosphere soil of *J. excelsa* (Crimea Peninsula, Ukraine). High-resolution LC-MS analysis of the crude extract of this strain revealed the presence of several peaks, two of which exhibited an interesting UV spectrum (Supplementary Materials Figure S1). These peaks were characterized by strong UV absorption signals at λ_max_ 226, 264, 300, and 408 nm and at λ_max_ 228, 278, 328, and 504 for peaks 1 and 2, respectively. Peak 1 had a retention time (tR) of 14.7 min and an m/z of 385.2005 Da [M+H]^+^ (**1**), whereas for peak 2 they were tR = 15.3 min and m/z 443.2187 Da [M+H]^+^ (**2**). The dereplication analysis of the identified monoisotopic masses 384.1929 (**1**) and 442.2114 (**2**) in the Dictionary of Natural Products Database (DNP) [14] yielded no matches. Thus, the lack of matches in the DNP database may indicate the novelty of these compounds.

To determine the structure of the identified compounds, the Je 1-369 strain was grown in 10 L of SG medium, and metabolites were extracted from the supernatant with ethyl acetate. The obtained extract was purified in three stages, including normal-phase chromatography on a silica gel column, size-exclusion chromatography through a Sephadex column, and preparative high-performance liquid chromatography (HPLC). As a result, 3.2 and 1.4 mg of compounds **1** and **2** were obtained.

**Figure 1:**
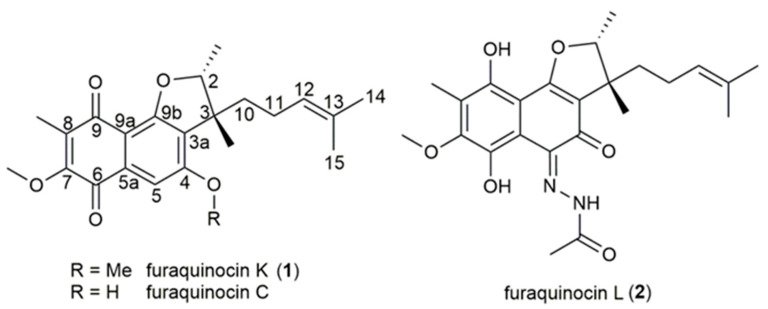
Furaquinocin C and the new furaquinocins K (**1**) and L (**2**).

The molecular formula of **1** was established as C_23_H_28_O_5_ based on the monoisotopic mass of m/z 385.2005 Da ([M + H]^+^). Its UV spectrum (Figure S1B) suggested a naphthoquinone moiety. ^1^H and ^13^C NMR together with DEPT-edited HSQC revealed 11 quaternary carbons, two methylenes, three methines, and 7 methyl groups, of which two belonged to a methoxy unit (δ_C_ 55.81, δ_H_ 3.95, 4-OMe, and δ_C_ 60.70, δ_H_ 4.00, 7-OMe). Careful analysis of the chemical shifts, 2D HHCOSY, and HMBC correlations (Table 1 and Figures S2–S7) led to a highly substituted 1,4-napthochinon and an unsaturated monoterpene unit. All these structural features revealed similarities to the known furaquinocins A-J [15,16,17]; this was especially observed with furaquinocin C, which, in contrast to **1**, lacked the methoxy group at C-4 (Figure 1). Therefore, we named **1** furaquinocin K, which is the first furaquinocin bearing a methoxy group at C-4.

Furaquinocin K contains two stereo centres at C-2 and C-3, which are in the 2R and 3R configuration in all naturally occurring furaquinocins [18]. To confirm this finding, the optical rotation value of furaquinocin K, [α]_D_^23^ = −78°, was compared with those of furaquinocin C (2*R*, 3*R*; [α]_D_^23^ = −25°) and its synthetic epimer 3-epifuraquinocin C (2*R*, 3*S*; [α]_D_^23^ = +58°) [19]. The negative value determined for furaquinocin K supported the expected 2*R*, 3*R* configuration.

Compound **2**, C_24_H_30_N_2_O_6_, m/z 443.2187 Da ([M + H]^+^), was present as a red solid. Its 1D and 2D NMR spectra (Table 2 and Figures S2 and S9–S14) revealed structural elements already known from furaquinocin K (**1**), including the 8-Me (δ_C_ 9.26 and δ_H_ 2.06) and 7-OMe (δ_C_ 60.70 and δ_H_ 4.00) groups and the monoterpene unit as part of a cyclic ether. However, in contrast to **1**, H-5 was missing, the five-membered ether ring was fused to a 1,2-benzoquinone derivative (δ_C_ 179.22 and 139.94) rather than a phenol, and the carbonyls of the former 1,4-benzoquinone moiety at C-6 and C-9 were converted into a hydroquinone moiety (δ_C_ 147.79 and 149.00). In addition, the molecular formula of **2** was enlarged by CH_2_N_2_O.

The nature of the two nitrogens was further analysed by 1H,15N-HSQC and 1H,15N-HMBC (Figures S15 and S16). This identified an NH signal at δN 171.7 and δH 14.90 and an N signal at δN 301.9, in which the chemical shifts matched a hydrazone unit. This was supported by the correlation of the NH proton with C-5 (δC 139.94) of the benzoquinone in the 1H,13C-HMBC. In addition, correlations of the NH proton and a methyl group (δC 22.12, δH 2.25 s, C-17) with the carbons of a carbonyl (δC 179.22, C-16) were detected, which indicated a directly adjacent acetyl group. Consequently, the structural component was assigned to an acetylhydrazone.

**Figure 2:**
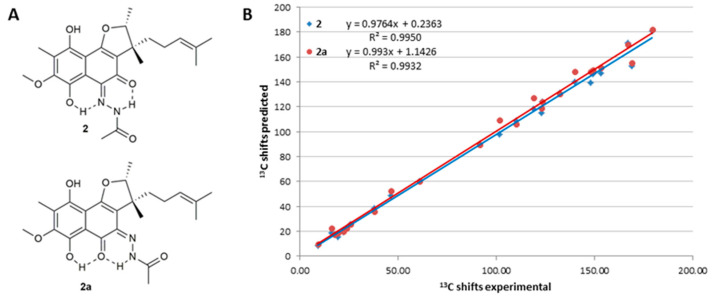
Comparison of the predicted 13C NMR shifts from ACD labs of the two possible furaquinocin L isomers **2** and **2a** (**A**) with the experimentally determined data (**B**).

The low-field-shifted proton signals of NH (δNH 14.90) and 6-OH (δOH 12.89) indicated intramolecular hydrogen bonds to the carbonyl oxygens and the imine nitrogen of hydrazine, respectively. This led to two possible constitutional isomers (**2** and **2a**) (Figure 2A), as C-4 and C-5 could not be distinguished by HMBC. To determine the correct isomer, the measured carbon shift of **2** was compared with predicted data for **2** and **2a**, which were calculated using the NMR prediction tool of ACD Labs, version 2021.2.0 (Figure 2B). As a result, the chemical shift values of isomer **2** showed a slightly better fit based on the regression coefficient values [20]. In addition, a 1H,15N-HMBC correlation from 6-OH to the imine nitrogen 5-N suggested a ^1h^J_N_, OH coupling caused by an O-H.... N intramolecular hydrogen bond, as also present in **2** [21], which strongly indicated that **2** was the correct structure.

### 2.2. Identification of the Furaquinocin Biosynthetic Gene Cluster

To identify the furaquinocin biosynthetic gene cluster, the genome of Je 1-369 was sequenced and analysed. The phylogenetic analysis based on the 16S rRNA gene sequence of the Je1–369 strain revealed this strain to belong to the genus *Streptomyces* (Figure S17). The total genome size of *Streptomyces* sp. Je 1-369 strain is 8,820,026 bp with 71% G+C. Terminal inverted repeats (TIRs) of 160,307 bp are present at the ends of the chromosome. The genome annotation of this strain identified 7695 probable protein-coding genes, eighteen rRNA genes in six operons, and eighty-seven tRNA genes. A preliminary analysis of the complete genome using antiSMASH showed the presence of thirty-six predicted gene clusters (two of which were duplicated in TIRs) (Supplementary Table S1). Among them, there were nine clusters of terpenes, five PKS, and three NRPS; others included lanthipeptide, siderophores, ectoine, phenazine, bacteriocin, butyrolactone, melanine, clusters, and several hybrid clusters. Two secondary metabolite gene clusters were located at the edges of the chromosome in the TIRs. One of them showed a 60% homology to the furaquinocin B (*fur*) biosynthetic gene cluster.

**Figure 3:**
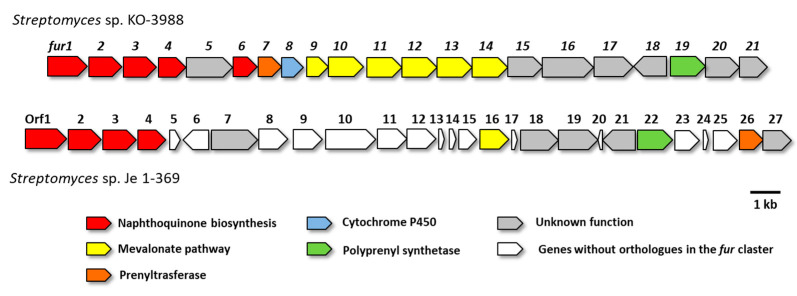
Comparison of the furaquinocin B biosynthetic gene clusters from *Streptomyces* sp. KO-3988 and *Streptomyces* sp. Je 1-369.

A detailed analysis of the detected cluster in *Streptomyces* sp. strain Je 1-369 showed its significant difference from the described *fur* cluster from *Streptomyces* sp. KO-3988. The main difference between the studied clusters lay in their gene cluster organization. However, the key furaquinocin-forming genes involved in naphthoquinone core formation (open reading frame (Orf) 1–4), polyprenyl synthetase (Orf22), and prenyltrasferase (Orf26) were presented. In addition, five of the six mevalonate pathway genes were missing from the identified cluster from the Je 1-369 strain, but these genes were detected outside the cluster. In addition, a complete set of genes (Orf8–Orf12) for the recycling of S-adenosylhomocysteine to S-adenosylmethionine were contained between the furaquinocine-forming genes in the cluster from the Je 1-369 strain (Figure 3 and Table 3). An identical gene arrangement occurred in the furanonaphthoquinone I gene cluster [22], which was a regioisomer of furaquinocin C.

### 2.3. Biological Activity of Novel Furaquinocin Analogues

The structures of the furaquinocin analogues identified in this study contained significant differences from those already described. Therefore, these differences in structure may have a significant effect on their activities. Thus, the identified furaquinocins K and L were tested against a wide range of test strains of bacteria, yeast, and fungi, including *Bacillus subtilis* DSM 10, *Staphylococcus aureus* Newman, *Mycobacterium smegmatis* mc2155, *Escherichia coli* BW25113 (wt), *E. coli* JW0451-2 (ΔacrB), *Pseudomonas aeruginosa* PA14, *Acinetobacter baumannii* DSM 30008, *Citrobacter freundii* DSM 30039, *Candida albicans* DSM 1665, *Cryptococcus neoformans* DSM 11959, *Pichia anomala* DSM 6766, and *Mucor hiemalis* DSM 2656. Furaquinocin K showed no antagonistic activity against the tested strains but demonstrated cytotoxicity against hepatocellular carcinoma (HepG2) cells with an IC_50_ value of 12.6 μg/mL. In turn, pure furaquinocin L showed antagonistic activity only against the Gram-positive bacteria *B. subtilis* DSM 10 (minimum inhibitory concentration (MIC) values of 64 μg/mL) and the *S. aureus* Newman strain (MIC values of 2 μg/mL) and no cytotoxicity against HepG2 cells at the maximum concentration (37 μg/mL) (Table 4).

## 3. Discussion

The numbers of cases involving antibiotic-resistant bacterial pathogens are increasing; as a result, mankind is facing a dilemma and screening for new antibiotics is urgently needed [23]. Microbial secondary metabolites are the predominant source of biologically active products that can be used as therapeutic agents to preserve human life and health [24]. Meroterpenoids can play a significant role in this endeavour, since most of them show an impressive range of biological activity [9]. In this study, we described the identification, purification, and structure elucidation of two new naphthoquinone-based meroterpenoids, furaquinocins K and L, from the *Streptomyces* sp. Je 1-369 strain isolated from the rhizosphere soil of *J. excelsa*. Furaquinocins are a small family of meroterpenoids, including furaquinocins A–J [15,16,17], furanonaphthoquinone I, which is a regioisomer of furaquinocin C [25], furaquinocin derivatives PI-220 [26], and JBIR-136 [27]. The furaquinocins A-J and their homologues differ only in the modification of the terpene side chains. However, the furaquinocins K and L isolated in this study contained a modification in the naphthoquinone moiety. In addition, the structure of furaquinocin L included an acetylhydrazone moiety, which is quite rare among natural products [28]. Not surprisingly, relatively little is known about hydrazone biosynthesis. A recent study showed that the hydrazone group is formed by nonenzymatic Japp–Klingemann coupling between the electrophilic diazotated alkyl 5-hydroxylanthranilate and a β-keto aldehyde-containing cyclic peptide precursor during tazicamide biosynthesis [29]. Given the cardinal difference in the structure between tazicamides and furaquinocin L, we believe that the formation of the hydrazone group between these structures will also be different.

The gene cluster of furaquinocin B was identified in the course of a study by Kawasaki et al. [30] due to its promising cytotoxic activity against human cancer cells. Genome analysis of the Je 1-369 strain revealed a gene cluster that showed a 60% homology with the cluster of furaquinocin B biosynthesis. Moreover, this cluster was located in the TIRs at the ends of the chromosome. The presence of gene clusters that are duplicated in streptomycete chromosomes is called “superclusters” and can often affect production levels [31]. However, we did not find any genes similar to the genes involved in N–N bond formation in the identified furaquinocin gene cluster. Thus, we suggest that extracluster genes or genes in which the function has not yet been characterized may be involved in the formation of the furaquinocin L structure. Thus, further studies on the biosynthesis of furaquinocin L, namely, the formation of acetylhydrazone, will clarify the nature and mechanisms of hydrazone-containing compound formation.

As mentioned above, the furaquinocins showed promising cytotoxic activity but did not exhibit antimicrobial activity. Furaquinocin K showed no antimicrobial activity but demonstrated cytotoxicity against HepG2 cells in the same manner as the described furaquinocins and their analogues. This was explained by the slight difference in the chemical structures, in which the difference between the C and K furaquinocins lay in the methoxy group at C-4 (Figure 1). We also hypothesized that the presence of acetylhydrazone in the structure of furaquinocin L may have a significant effect on its activity, since N–N-containing natural compounds exhibit a diverse spectrum of biological activities [28]. As a result, furaquinocin L containing acetylhydrazone showed activity against Gram-positive bacteria in the absence of cytotoxicity. Therefore, to the best of our knowledge, this is the first furaquinocin with antibacterial activity. Thus, our study expands the possibilities of using furaquinocins not only as anticancer agents but also as potential antibacterial agents.

## 4. Materials and Methods

### 4.1. General Experimental Procedures

The strain Je 1-369 isolated from the rhizosphere soil of *J. excelsa* was used in this study. This strain was isolated by direct inoculation of an aqueous suspension of soil on OM medium (20 g/L oat flour and 20 g/L agar; pH 7.2) and incubated at 28 °C for 14 days. Spores and mycelial suspensions of Je 1-369 strain were stored in 20% (*v*/*v*) glycerol solution at −20 °C and deposited in the Microbial Culture Collection of Antibiotic Producers (MCCAP) of Ivan Franko National University of Lviv (collection number Lv 391).

OM medium was used to cultivate the actinomycete strain. Liquid tryptic soy broth (TSB) (Sigma-Aldrich, St. Louis, MO, USA) was used for the extraction of total DNA. SG medium (20.0 g/L glucose, 10 g/L soy peptone, and 2 g/L CaCO_3_; pH 7.2) was used to produce the secondary metabolites.

### 4.2. Secondary Metabolite Extraction and Analysis

To extract the secondary metabolites, the *Streptomyces* sp. Je 1-369 strain was grown in 15 mL of TSB in a 100 mL flask for 2 days at 28 °C and 180 rpm, and 1 mL of pre-culture was inoculated into 100 mL of production SG medium in a 500 mL flask. The Je 1-369 strain was grown for 7 days at 28 °C and 180 rpm in an Infors multitron shaker (Infors AG, Basel, Switzerland). The secondary metabolites of the Je 1-369 strain were then extracted from the culture supernatant with an equal amount of ethyl acetate and acetone:methanol (1:1) mixture from the culture biomass. The obtained extracts were evaporated using an IKA RV-8 rotary evaporator (IKA, Staufen, Germany) at 40 °C and were dissolved in methanol. The extracts were analysed on a Dionex Ultimate 3000 UPLC system (ThermoFisher Scientific, Waltham, MA, USA) coupled to a PDA detector using a 100 mm ACQUITY UPLC BEH C18 1.7 μm column (Waters Corporation, Milford, MA, USA). A linear gradient (5% to 95% solvent B) of a water solution containing 0.1% (*v*/*v*) formic acid (solvent A) and an acetonitrile solution containing 0.1% (*v*/*v*) formic acid (solvent B) as the mobile phase was used to separate the extracts at a flow rate of 0.6 mL/min for 18 min. Mass analysis was performed on a Bruker Amazon Speed (Bruker, Billerica, MA, USA) mass spectrometer using the positive mode of ionization and a range detection of 150–2000 m/z. Data were analysed using the Compass Data Analysis v. 4.2 (Bruker, Billerica, MA, USA).

### 4.3. Secondary Metabolite Purification

Secondary metabolites were extracted from the *Streptomyces* sp. Je 1-369 strain grown in 10 L of SG medium. The ethyl acetate extract from the culture supernatant of this strain was dissolved in methanol and purified in three stages. The first purification stage was normal-phase chromatography on a silica gel column with hexane (solvent A), chloroform (solvent B), ethyl acetate (solvent C), and methanol (solvent D) as the mobile phase at a flow rate of 100 mL/min. A triple linear gradient of each solvent pair A/B (10 column volumes (CV)), B/C (15 CV) and C/D (15 CV) was used, and fractions were collected every 18 mL. The separation was performed on a Biotage Isolera One LC-system (Biotage, Uppsala, Sweden) using a SNAP Ultra 50 g column HP-Sphere (Biotage, Uppsala, Sweden). The fractions containing the compounds of interest were pooled together, concentrated, and used for the second separation stage, which was size-exclusion chromatography on a Sephadex LH-20 column (Sigma-Aldrich, St. Louis, MO, USA) with methanol as the mobile phase. The fractions containing the compound of interest were again pooled together and concentrated. The last purification stage was reversed-phase high-performance liquid chromatography (HPLC), separation on a semipreparative C18 column SynergiTM 4 μm Fusion-RP 80 Å 250 mm × 10 mm (Phenomenex, Torrance, CA, USA) using a water solution containing 0.1% (*v*/*v*) formic acid (solvent A), and an acetonitrile solution containing 0.1% (*v*/*v*) formic acid (solvent B) as a mobile phase. The following gradient at a flow rate of 4 mL/min was used for compound **1** separation: 0 min—35% B, 0.5 min—35%, 2.5 min—70% B, 17.5 min—95% B, 21 min—95% B, 22 min—35% B, and 23 min—35% B. The following gradient at a flow rate of 4 mL/min was used for compound **2** separation: 0 min—35% B, 0.5 min—35%, 2.5 min—50% B, 17.5 min—95% B, 21 min—95% B, 22 min—35% B, and 25 min—35% B. The fractions containing pure compound were pooled together and evaporated. For compound **1** fractions with tR between 16.0 and 17.0 min were collected and for compound **2** fractions with tR between 20.2 and 20.8 min were collected. The quality assessment of the purification at each stage was verified by HPLC–MS.

### 4.4. Nuclear Magnetic Resonance (NRM) Spectroscopy and Optical Rotation (OD)

The NMR spectra of furaquinocin K were recorded on a Bruker Avance I UltraShield 500 MHz (Bruker, BioSpin GmbH, Rheinstetten, Germany) equipped with a 5 mm BBO probe at 298 K. The NMR spectra of furaquinocin L were acquired on a Bruker Avance III Ascent 700 MHz spectrometer at 298 K equipped with a 5 mm TCI cryoprobe. The chemical shifts (δ) were reported in parts per million (ppm) relative to TMS. As solvents, CDCl_3_ (δ_H_ 7.27 ppm, δ_C_ 77.00 ppm) and DMSO-*d*_6_ (δ_H_ 2.50 ppm, δ_C_ 39.51 ppm, 1 drop TFA) from Deutero (Kastellaun, Germany) were used. DEPT-edited ^1^H,^13^C-HSQC, ^1^H,^13^C-HMBC, ^1^H-^1^H COSY, ^1^H,^15^N-HSQC, and ^1^H,^15^N-HMBC spectra were recorded using standard pulse programs from TOPSPIN v.3.6. Optical rotations were measured using a JASCO P-2000 digital polarimeter (28600 Mary’s Ct, Easton, MD, USA).

### 4.5. Antimicrobial Susceptibility Test and Cytotoxicity Assay

MICs were determined according to standard procedures. Single colonies of the bacterial strains were suspended in cation-adjusted Müller–Hinton broth to obtain a final inoculum of 10^4^ CFU mL^−1^. Serial dilutions of furaquinocins (0.5 to 64 µg/mL) were prepared in sterile 96-well plates, and the bacterial suspension was added. Growth inhibition was assessed after overnight incubation at 30–37 °C for 16–18 h. A total of 5 μL of thiazolyl blue tetrazolium bromide (MTT, 10 mg/mL) solution was added to each well and plates were incubated at 30 °C for 1 h. MICs were evaluated visually as the concentration of furaquinocins in a well, in which the compound colour did not change from yellow to dark blue. The following microbial test cultures were used: *B. subtilis* DSM 10, *S. aureus* Newman, *M. smegmatis* mc2155, *E. coli* BW25113 (wt), *E. coli* JW0451-2 (ΔacrB), *P. aeruginosa* PA14, *A. baumannii* DSM 30008, *C. freundii* DSM 30039, *C. albicans* DSM 1665, *C. neoformans* DSM 11959, *P. anomala* DSM 6766, and *M. hiemalis* DSM 2656.

The HepG2 cell line was used to evaluate the cytotoxic activity of the isolated furaquinocins. The cell line was obtained from the German Collection of Microorganisms and Cell Cultures (Deutsche Sammlung für Mikroorganismen und Zellkulturen (DSMZ)) and cultured under the conditions recommended by the depositor. Cells were grown and diluted to 5 × 10^4^ per well of 96-well plates in 180 μL of complete RPMI-1640 medium (+10% fetal bovine serum (FBS)). After 2 h of equilibration (37 °C and 5% CO_2_), the cells were treated with a serial dilution of furaquinocins in methanol. A total of 20 μL of 5 mg/mL MTT in phosphate-buffered saline (PBS) was added to each well after the cells were grown for 5 days (37 °C and 5% CO_2_). The cells were further incubated for 2 h at 37 °C before the supernatant was discarded. Subsequently, the cells were washed with 100 μL of PBS and treated with 100 μL of 2-propanol/10 N HCl (250:1) to dissolve formazan granules. Cell viability was measured as a percentage relative to the respective methanol control by measuring the absorbance at 570 nm with a microplate reader (Tecan Infinite 200 PRO). GraphPad Prism was used for sigmoidal curve fitting to determine the IC50 values as well as the calculation of confidence intervals.

### 4.6. Genome Sequencing and Bioinformatics Analysis

To extract the total DNA, the *Streptomyces* sp. Je 1-369 strain was grown in TSB medium for four days at 28 °C with a shaking rate of 180 rpm. A salting-out procedure was used to obtain the total DNA [32]. The RNA-free genomic DNA of strain Je 1-369 was sequenced using an Illumina paired-end sequencing library (TruSeq sample preparation kit; Illumina, USA) as recommended by the manufacturer. The Illumina sequencing data were de novo assembled using Newbler v2.8 (454 Life Sciences, Branford, CT, USA) with default settings. Gene prediction and genome annotation were carried out using the prokka v1.11 and GenDB 2.0 platforms [33,34]. The secondary metabolite gene clusters were analysed using the antiSMASH genome mining tool [35]. The analysis of genetic data was performed using Geneious 9.1.2 software [36]. The whole genome sequences of *Streptomyces* sp. Je 1-369 were deposited under accession number CP101750 (PRJNA860344 for BioProject and SAMN29841164 for BioSample) into the GenBank database.

## 5. Conclusions

In summary, two new naphthoquinone-based meroterpenoids, furaquinocin K and L, were isolated from the *Streptomyces* sp. Je 1-369 strain. Their structures were elucidated by NMR and found to contain modifications in the polyketide naphthoquinone skeleton that have not yet been described for furaquinocins. Deciphering the biosynthesis of these furaquinocin analogues will expand the knowledge about the biosynthesis of meroterpenoids as well as the formation of hydrazones in natural compounds.

## Figures and Tables

**Table 1:** NMR chemical shifts and 2D correlations of furaquinocin K recorded in CDCl_3_.

Position	^a^ δ_C_, Type	^b^ δ_H_, Mult. (J in Hz)	COSY	^c^ HMBC
2	87.72, CH	4.84, q (6.6)	16	3, 3a, 9b, 10, 2-Me, 3-Me
2-Me	15.32, CH3	1.46, d (6.6)	2	2, 3
3	47.12, C	-	-	-
3-Me	19.79, CH3	1.22, s	-	2, 3, 3a, 10
3a	128.99, C	-	-	-
4	159.77, C	-	-	-
4-OMe	55.81, CH3	3.95, s	-	4, 5
5	103.30, CH	7.21, s	-	3, 3a, 4, 5a, 6,9, 9a
5a	133.58, C	-	-	-
6	180.98, C	-	-	-
7	156.80, C	-	-	-
7-OMe	60.70, CH3	4.00, s	-	7
8	133.31, C	-	-	-
8-Me	9.26, CH3	2.06, s	-	6, 7, 8, 9, 7-OMe
9	184.10, C	-	-	-
9a	110.20, C	-	-	-
9b	160.23, C	-	-	-
10	37.61, CH2	1.93, m 1.59, dd (8.0, 11.9)	11	2, 3, 3a, 11, 12, 3-Me
11	23.71, CH2	1.94, m 1.77, m	10, 12	3, 10, 12, 13
12	123.82, CH	5.06, m	11, 14, 15	10, 11, 14, 15
13	131.90, C	-	-	-
14	25.65, CH3	1.66, bs	12, 15	12, 13, 15
15	17.49, CH3	1.53, bs	12, 14	12, 13, 14

^a^ 125 MHz for ^13^C-NMR. ^b^ 500 MHz for ^1^H-NMR. ^c^ HMBC correlations from protons to the indicated carbons.

**Table 2:** NMR chemical shifts and 2D correlations of furaquinocin L recorded in CDCl_3_.

Position	^a^ δ_C_, Type	^b^ δ_H_, Mult. (J in Hz)	COSY	^c^ HMBC
2	91.43, CH	4.99, q (6.5)	2-Me	3-Me, 10, 9b
2-Me	15.83, CH3	1.5, d (6.7)	2	2, 3
3	46.41, C	-	-	-
3-Me	19.21, CH3	1.32, s	-	2, 3, 3a, 10
3a	118.74, C	-	-	-
4	179.22, C	-	-	-
5	139.94, C	-	-	-
5-N	^d^ 301.9, N	-	-	-
5a	109.99, C	-	-	-
6	147.79, C	-	-	-
6-OH	-	12.89, s	-	5a, 6, 7, ^d^ 5-N
7	153.15, C	-	-	-
7-OMe	60.77, CH	3.99, s	-	7
8	123.08, C	-	-	-
8-Me	9.10, CH3	2.22, s	-	8, 7, 9
9	149.00, C	-	-	-
9-OH	-	8.10, s	-	7, 8, 9, 9a
9a	101.44, C	-	-	-
9b	169.10, C	-	-	-
10	37.91, CH2	1.72, m 1.94, m	11	3, 11
11	23.55, CH2	1.87, m 2.01, m	10, 12	11, 14, 15
12	123.49, CH	5.09, t (7.37)	11, 14, 15	10, 12
13	132.20, C	-	-	-
14	25.66, CH3	1.66, s	12, 15	12, 15
15	17.70, CH3	1.56, s	12, 14	12, 14
16	167.03, CO	-	-	-
16-NH	^d^ 171.7, NH	14.90, s		5, 16
17	22.12, CH3	2.25, s	-	16, ^d^ 16-NH

^a^ 125 MHz for ^13^C-NMR. ^b^ 500 MHz for ^1^H-NMR. ^c^ HMBC correlations from protons to the indicated carbons. ^d^ Determined from ^1^H,^15^N-HSQC, and ^1^H,^15^N-HMBC experiments in DMSO containing one drop of TFA.

**Table 3:** Identified ORFs, their putative gene functions, and their respective homologs in the furaquinocin (*fur*) biosynthetic gene cluster.

Gene in Je 1-369	ORFs * from *fur* Gene Cluster		Putative Product and Their Accession Number (% Identity) **
	Gene	% Identity/Coverage	
Orf1	*fur1*	81/99	type III polyketide synthase of *Streptomyces* sp. SID10115 (98.0%), A0A6B2SFW1
Orf2	*fur2*	81/88	cupin-domain-containing protein of *Streptomyces* sp. SID10115 (93.8%), A0A6B2SAS0
Orf3	*fur3*	81/93	aminotransferase of *Streptomyces* sp. SID10115 (93.8%), WP_150181863
Orf4	*fur4*	74/88	methyltransferase of *Streptomyces* sp. SID10115 (86.8%), A0A6B2SP85
Orf5			NAD(P)H:quinone oxidoreductase of *Streptomyces* sp. SID10115 (96.0%), A0A6B2SAG1
Orf6			enoyl-CoA hydratase/isomerase of *Frankia* sp. EUN1f (32.5%), D3CRF9
Orf7	*fur5*	73/98	acyl-CoA ligase of *Streptomyces* sp. SID10115 (95.3%), A0A6B2SZ49
Orf8			S-adenosylmethionine synthase of *Streptomyces* sp. SID10115 (97.5%), A0A6B2SM86
Orf9			carbohydrate kinase of *Streptomyces* sp. SID10115 (92.0%), A0A6B2SJJ8
Orf10			methionine synthase of *Streptomyces* sp. SID10115 (92.5%), A0A6B2SFQ1
Orf11			methylenetetrahydrofolate reductase of *Streptomyces* sp. SID10115 (93.4%), A0A6B2ST43
Orf12			adenosylhomocysteinase of *Streptomyces* sp. SID10115 (94.9%), A0A6B2SGN4
Orf13			surface protein of *Streptomyces* sp. SID10115 (68%), A0A6B2SEN8
Orf14			TetR_C_16-domain-containing protein of *Streptomyces* sp. SID10115 (92.3%), A0A6B2SJP1
Orf15			MFS transporter of *Streptomyces* sp. SID10115 (96.1%), A0A6B2SIA2
Orf16	*fur9*	68/93	mevalonate kinase of *Streptomyces* sp. SID10115 (75.0%), A0A6B2SSL0
Orf17			uncharacterized protein of *Streptomyces* sp. SID10115 (52.2%), A0A6B2SMM5
Orf18	*fur16*	72/92	FAD-binding protein of *Streptomyces* sp. Ru71 (72.2%), A0A2S4YSV4
Ofr19	*fur17*	75/92	3-carboxy-cis, cis-muconate cycloisomerase of *Streptomyces* sp. Ru71 (77.8%), A0A2S4YT60
Orf20			helix–turn–helix-domain-containing protein of *Streptomyces* sp. SID10116 (100%), A0A6B2SPW8
Orf21	*fur18*	54/99	uncharacterized protein of *Streptomyces* sp. SID10115 (94.7%) A0A6B2ST33
Orf22	*fur19*	71/98	polyprenyl synthetase family protein of *Streptomyces* sp. SID10115 (95.5%), A0A6B2SPK4
Orf23			FAD-binding protein of *Streptomyces* sp. SID10115 (90.5%), A0A6B2STJ0
Orf24			pyridine nucleotide-disulfide oxidoreductase of Streptomyces sp. MZ04 (45.5%), A0A4R9EYD3
Orf25			cytochrome bc1 complex cytochrome b subunit of *Streptomyces* sp. SID10115 (94.3%), A0A6B2SYI1
Orf26	*fur7*	66/98	prenyltransferase of *Streptomyces* sp. SID10115 (92.7%), A0A6B2SAL2
Orf27	*fur21*	61/99	Methyltransferase-domain-containing protein of *Streptomyces* sp. SID10115 (96.8%), A0A6B2SFQ2

* Open reading frame. ** Best match found by UniProt Protein–Protein BLAST.

**Table 4:** Antibacterial and cytotoxic activities of furaquinocins K and L.

Test Strain/Cell Line	Furaquinocin K (MIC, μg/mL)	Furaquinocin L (MIC, μg/mL)
*Bacillus subtilis* DSM 10	>64	64
*Staphylococcus aureus* Newman	>64	2
*Mycobacterium smegmatis* mc2155	>64	>64
*Escherichia coli* BW25113 (wt)	>64	>64
*E. coli* JW0451-2 (ΔacrB)	>64	>64
*Pseudomonas aeruginosa* PA14	>64	>64
*Acinetobacter baumannii* DSM 30008	>64	>64
*Citrobacter freundii* DSM 30039	>64	>64
*Candida albicans* DSM 1665	>64	>64
*Cryptococcus neoformans* DSM 11959	>64	>64
*Pichia anomala* DSM 6766	>64	>64
*Mucor hiemalis* DSM 2656	>64	>64
HepG2	12.6	>37

## Data Availability

Not applicable.

## Supplementary Materials

The following supporting information can be downloaded at: https://www.mdpi.com/article/10.3390/antibiotics11111587/s1, Figure S1: HPLC-MS analysis of crude extract of Streptomyces sp. Je 1-369. (**A**) Base peak chromatograms of crude extract, peaks corresponding to furaquinocin K and L are indicated by 1 and 2, respectively. (**B**) Mass spectrum (MS) and UV spectrum (UV) of furaquinocin K. (**C**) Mass spectrum (MS) and UV spectrum (UV) of furaquinocin L; Figure S2: Identified furaquinocins. (**A**) Structure of furaquinocin K (1) and furaquinocin L (2). (**B**) Selected COSY and HMBC correlations for furaquinocin K (1) and L (2); Figure S3: ^1^H-NMR spectrum (500 MHz, CDCl_3_) of furaquinocin K; Figure S4: ^13^C-NMR spectrum (125 MHz, CDCl_3_) of furaquinocin K; Figure S5: ^1^H-^1^H-COSY spectrum (CDCl_3_) of furaquinocin K; Figure S6: Edited HSQC spectrum (CDCl_3_) of furaquinocin K; Figure S7: ^1^H,^13^C-HMBC spectrum (CDCl_3_) of furaquinocin K; Figure S8: NOESY spectrum (CDCl3) of furaquinocin K; Figure S9: ^1^H-NMR spectrum (500 MHz, CDCl_3_) of furaquinocin L; Figure S10: ^13^C-NMR spectrum (125 MHz, CDCl_3_) of furaquinocin L; Figure S11: ^1^H-^1^H-COSY spectrum (CDCl_3_) of furaquinocin L; Figure S12: Edited HSQC spectrum (CDCl_3_) of furaquinocin L; Figure S13: ^1^H,^13^C-HMBC spectrum (CDCl_3_) of furaquinocin L; Figure S14: HMBC spectrum (CDCl_3_) of furaquinocin L with impurities but higher quantity; Figure S15: ^1^H,^15^N-HSQC spectrum (DMSO-d_6,_ TFA) of furaquinocin L; Figure S16: ^1^H,^15^N-HMBC spectrum (DMSO-d_6,_ TFA) of furaquinocin L; Figure S17: NJ tree based on the 16S rRNA gene sequences of the Je1–369 (in bold), its closest neighbours and selected Streptomyces-type strains. Micromonospora echinospora DSM 43816 was used as an outgroup; Table S1: Secondary metabolite biosynthesis gene clusters identified in the genome of Streptomyces sp. Je 1-369.

## Sample Availability

Samples of the compounds are available from the authors.
