# Supplementary material for: Furaquinocins K and L: Novel Naphthoquinone-Based Meroterpenoids from Streptomyces sp. Je 1-369
Source: Antibiotics (Basel). 2022 Nov 10;11(11):1587. doi: 10.3390/antibiotics11111587 (PMC9686526; doi:10.3390/antibiotics11111587)
Supplement: Supplementary file 1 [file antibiotics-11-01587-s001.zip › antibiotics-2005604-supplementary.pdf]

Supplementary Information

Furaquinocins K and L: novel naphthoquinone-based mero-terpenoids from Streptomyces sp. Je 1-369

Stepan Tistechok [1], Marc Stierhof [2], Maksym Myronovskyi [2], Josef Zapp [3], Oleksandr Gromyko [1,4] and Andriy Luzhetskyy [2,5*]

1 Department of Genetics and Biotechnology, Ivan Franko National University of Lviv, 79005 Lviv, Ukraine
2 Department of Pharmaceutical Biotechnology, Saarland University, 66123 Saarbruecken, Germany
3 Department of Pharmaceutical Biology, Saarland University, 66123 Saarbruecken, Germany
4 Microbial Culture Collection of Antibiotic Producers, Ivan Franko National University of Lviv, 79005 Lviv, Ukraine
5 Helmholtz Institute for Pharmaceutical Research Saarland, 66123 Saarbruecken, Germany
* Correspondence: a.luzhetskyy@mx.uni-saarland.de; Tel.: +49-681-302-70200 (A.L.)

Physical data of new furaquinocins:

furaquinocin K (1). yellow solid; 3.2 mg; $[\alpha]_D^{20}$ -78°; (c 0.12, MeOH); UV (73 % ACN in H_2O + 0.1% FA) λ_{max} (log ε) 226 nm (2.31), 264 (1.55), 300 (2.25) and 408 nm (1.04); ^1H and ^{13}C NMR data, see Table 1; ESI-TOF-MS m/z 385.2005 [M+H]$^+$ (calc. for $C_{23}H_{29}O_5$ 385.2015).

furaquinocin L (2). red solid; 1.4 mg; UV (76% ACN in H_2O + 0.1% FA) λ_{max} (log ε) 228 nm (2.36), 278 nm (1.74), 328 nm (1.79) and 504 nm (1.59); ^1H and ^{13}C NMR data, see Table 2; ESI-TOF-MS m/z 443.2187 [M+H]$^+$ (calc. for $C_{24}H_{31}N_2O_6$ 443.21821).

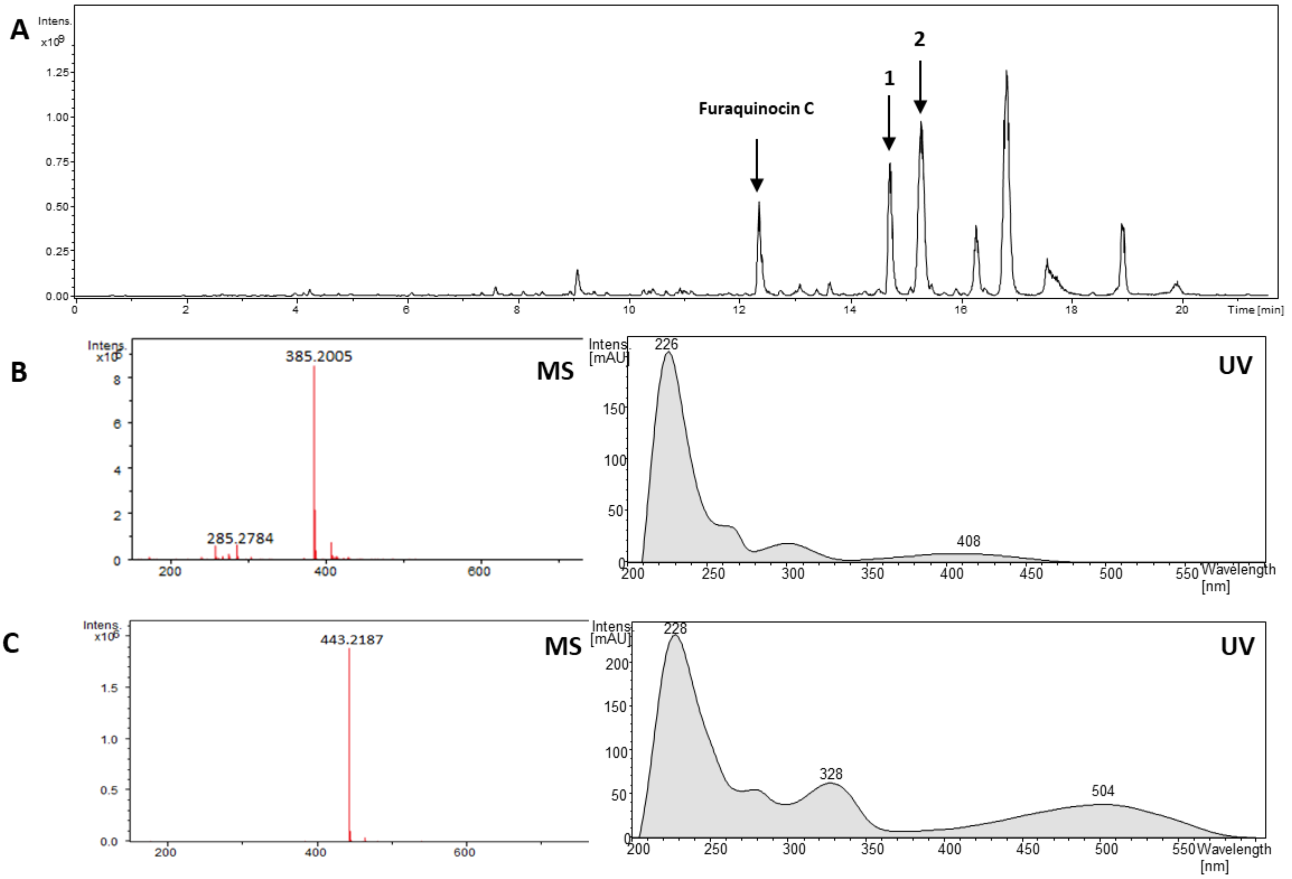

Figure S1: HPLC-MS analysis of crude extract of Streptomyces sp. Je 1-369. (**A**) Base peak chromatograms of crude extract, peaks corresponding to furaquinocin K and L are indicated by 1 and 2, respectively. (**B**) Mass spectrum (MS) and UV spectrum (UV) of furaquinocin K. (**C**) Mass spectrum (MS) and UV spectrum (UV) of furaquinocin L.

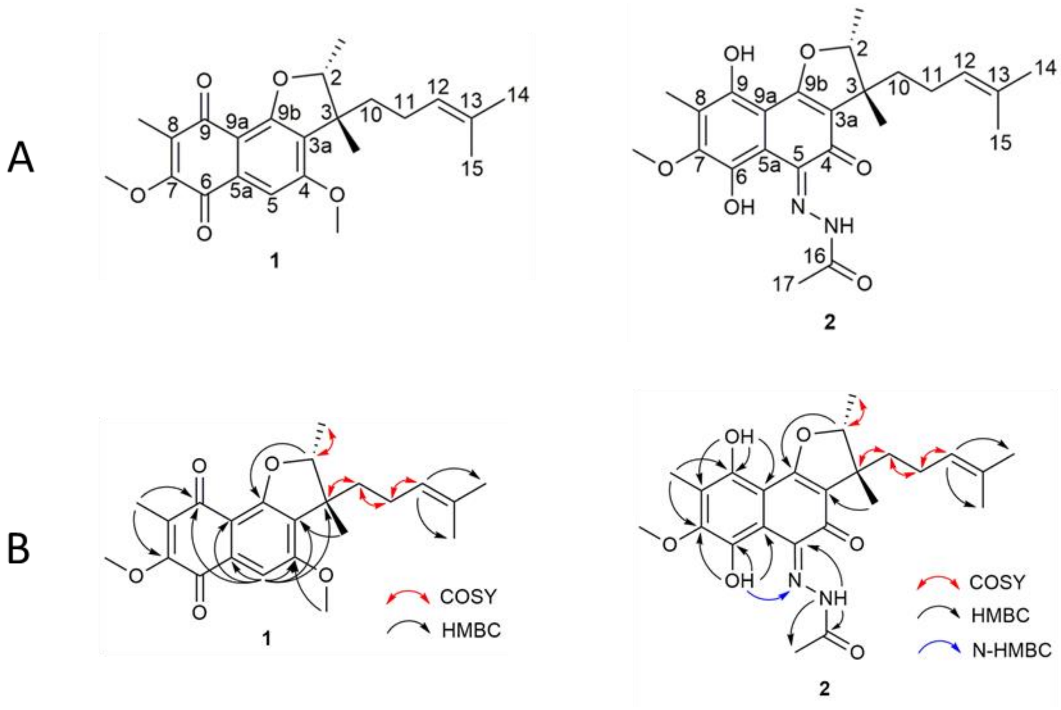

Figure S2: Identified furaquinocins. (**A**) Structure of furaquinocin K (1) and furaquinocin L (2), (**B**) Selected COSY and HMBC correlations for furaquinocin K (1) and L (2).

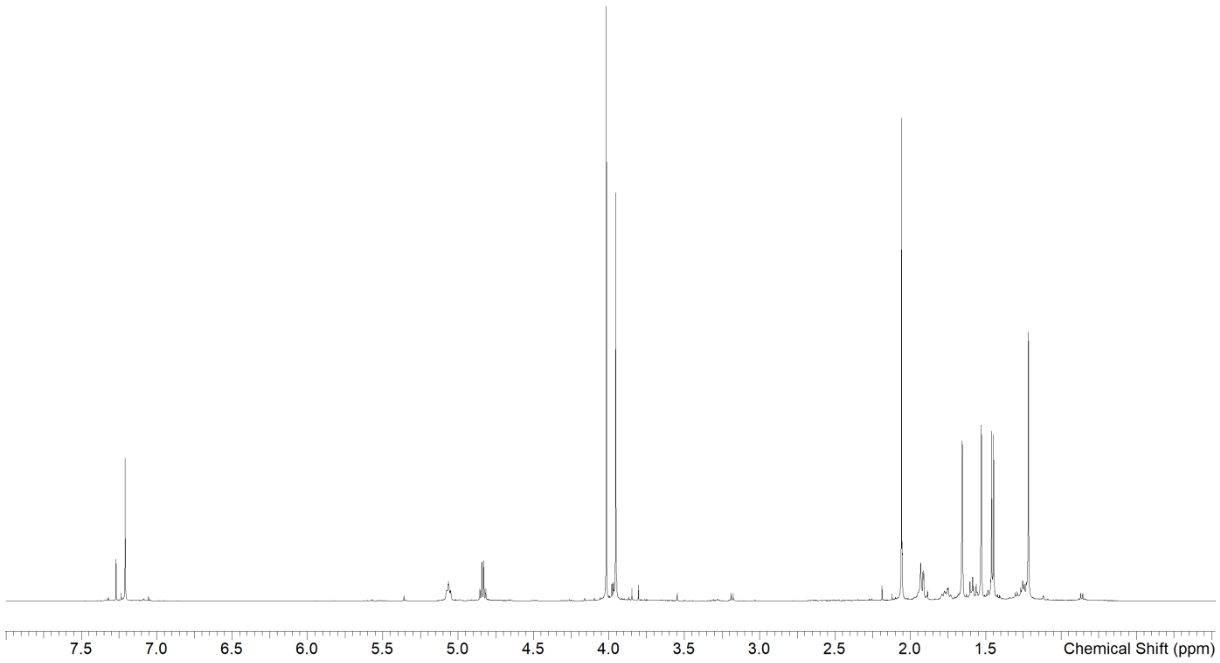

Figure S3: ^{1}H-NMR spectrum (500 MHz, $CDCl_3$) of furaquinocin K.

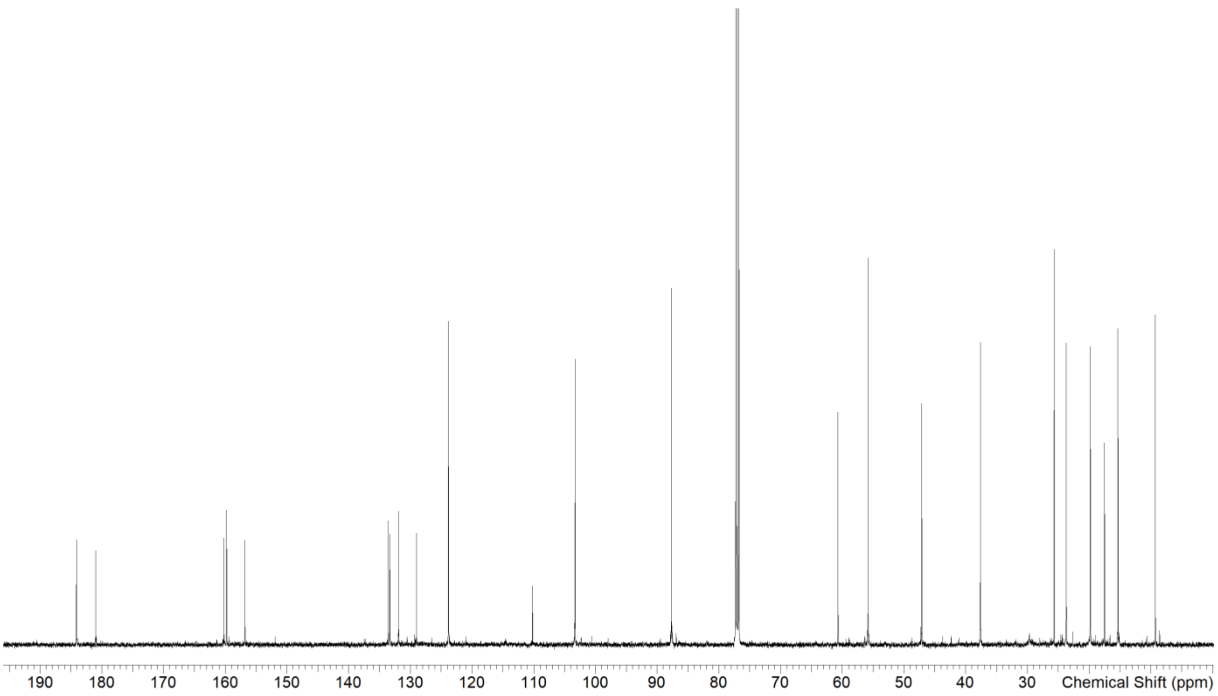

Figure S4: ^{13}C-NMR spectrum (125 MHz, $CDCl_3$) of furaquinocin K.

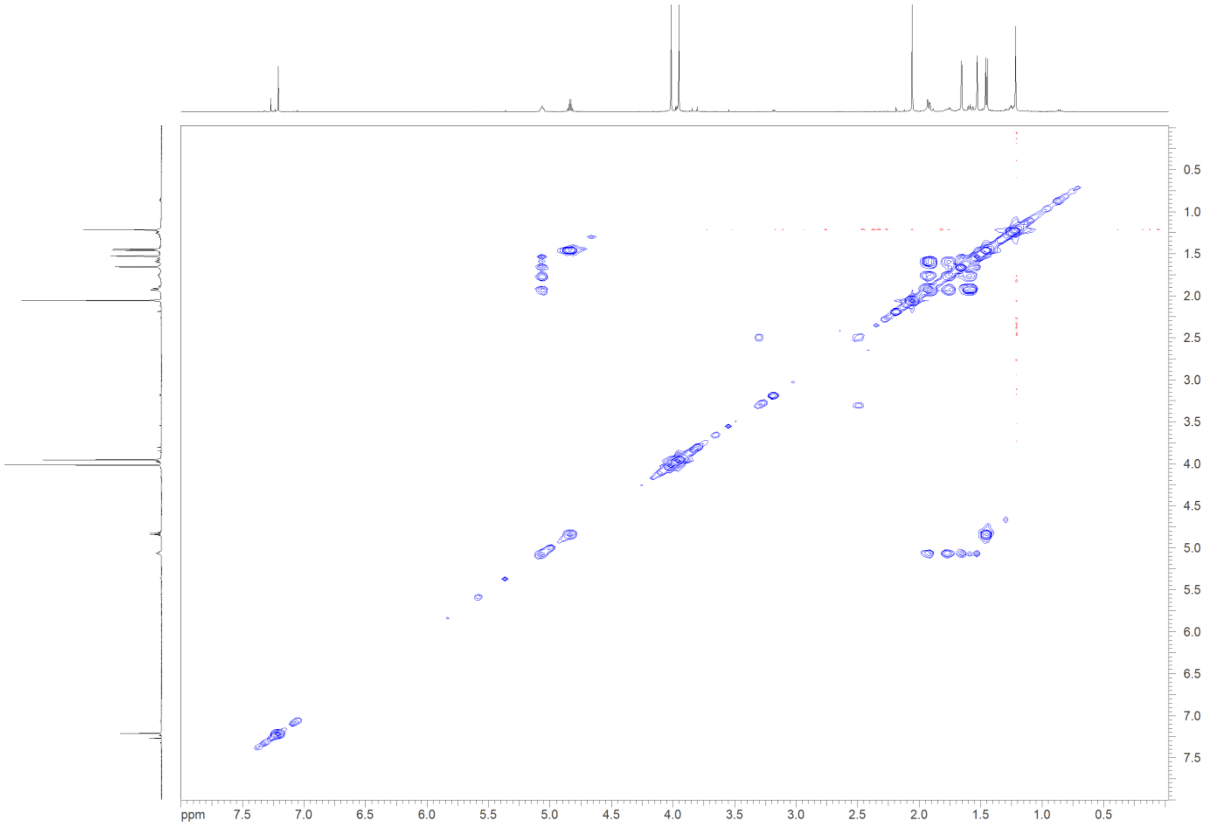

Figure S5: ^{1}H-^{1}H-COSY spectrum ($CDCl_3$) of furaquinocin K.

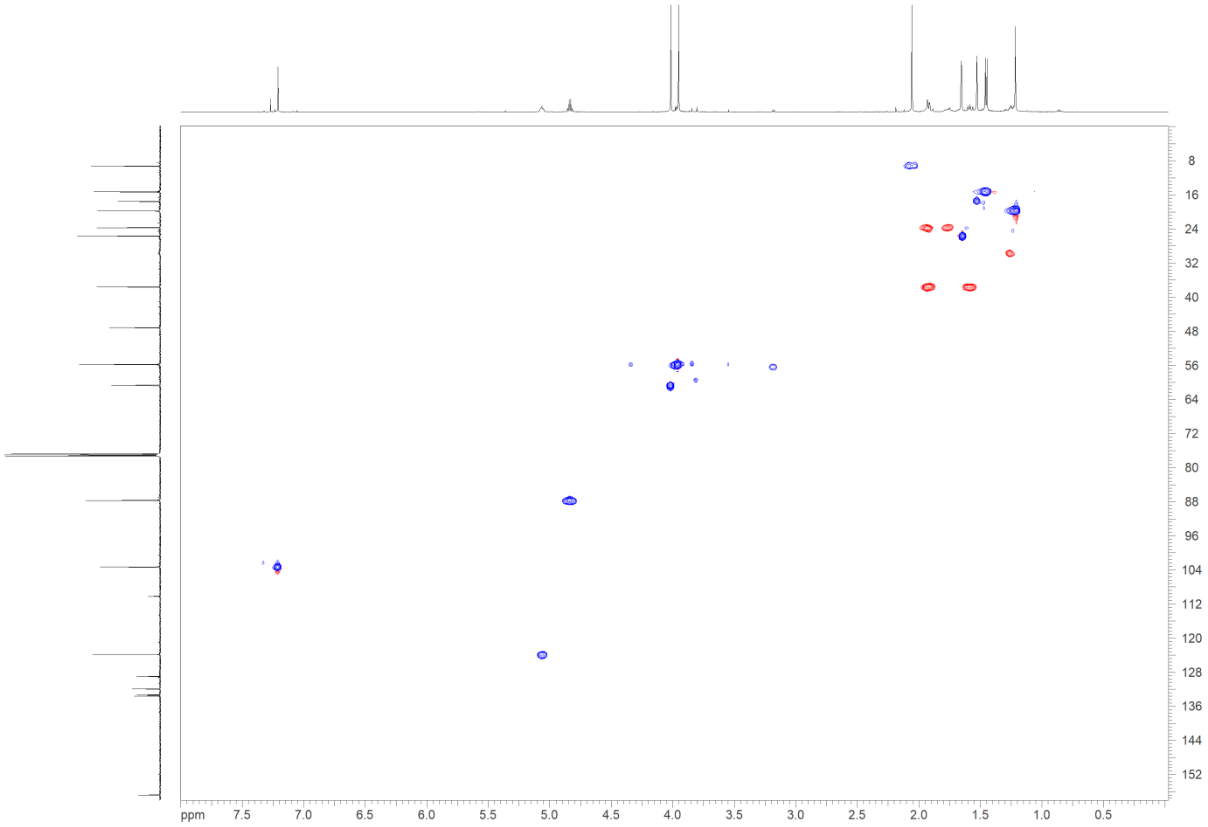

Figure S6: Edited HSQC spectrum ($CDCl_3$) of furaquinocin K.

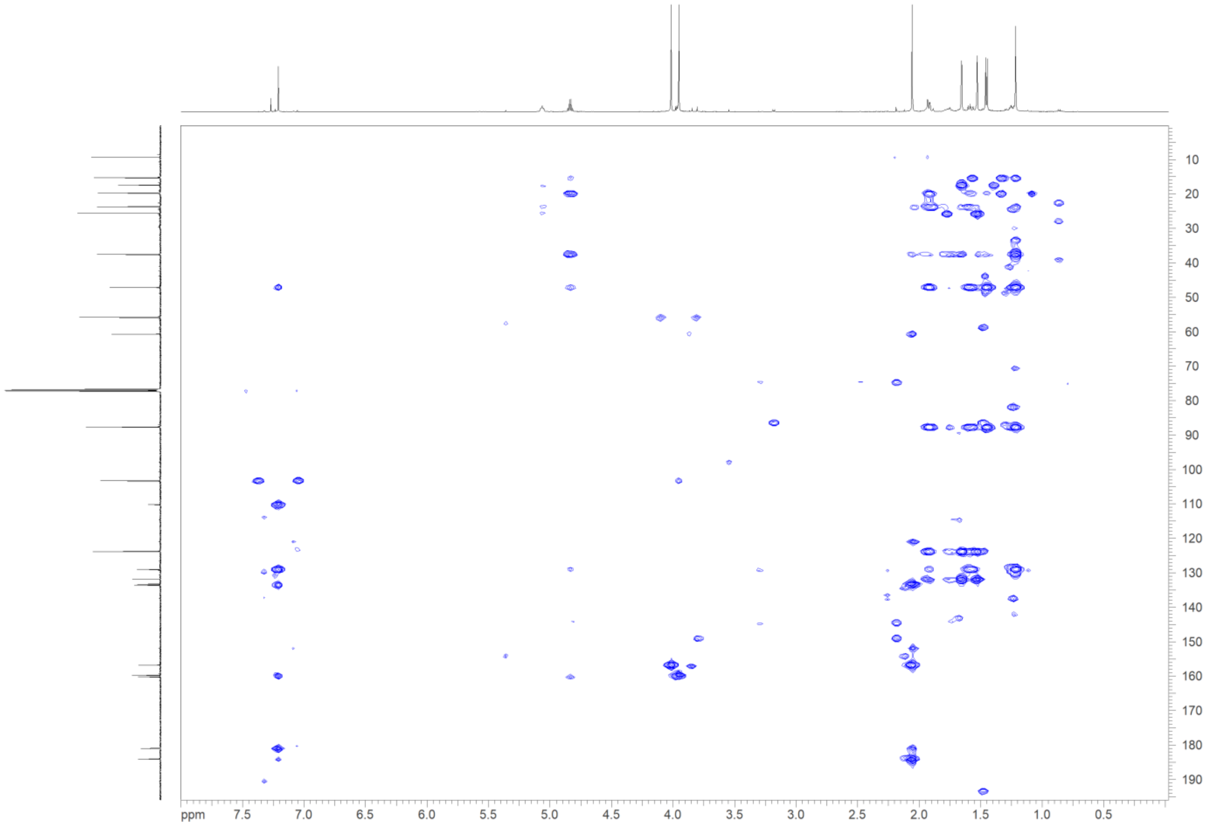

Figure S7: $^1H,^{13}C$-HMBC spectrum ($CDCl_3$) of furaquinocin K.

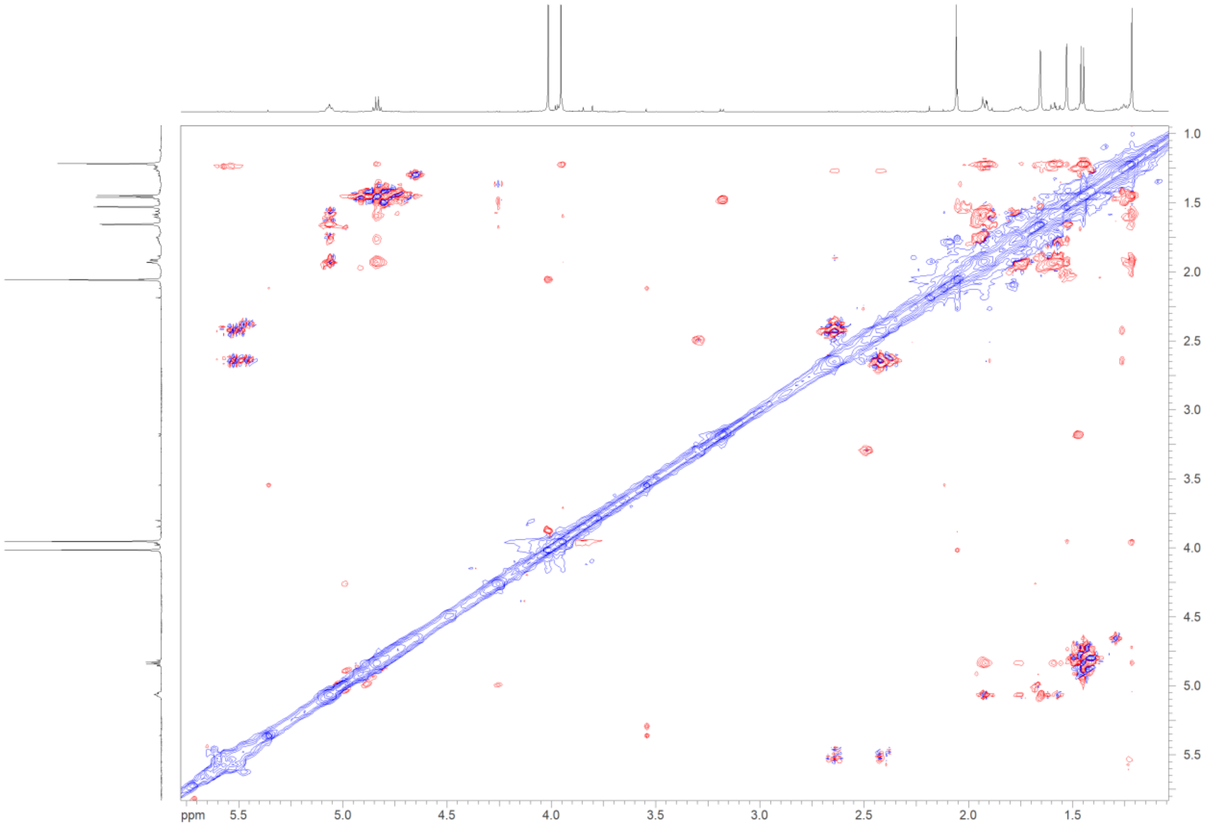

Figure S8: NOESY spectrum ($CDCl_3$) of furaquinocin K.

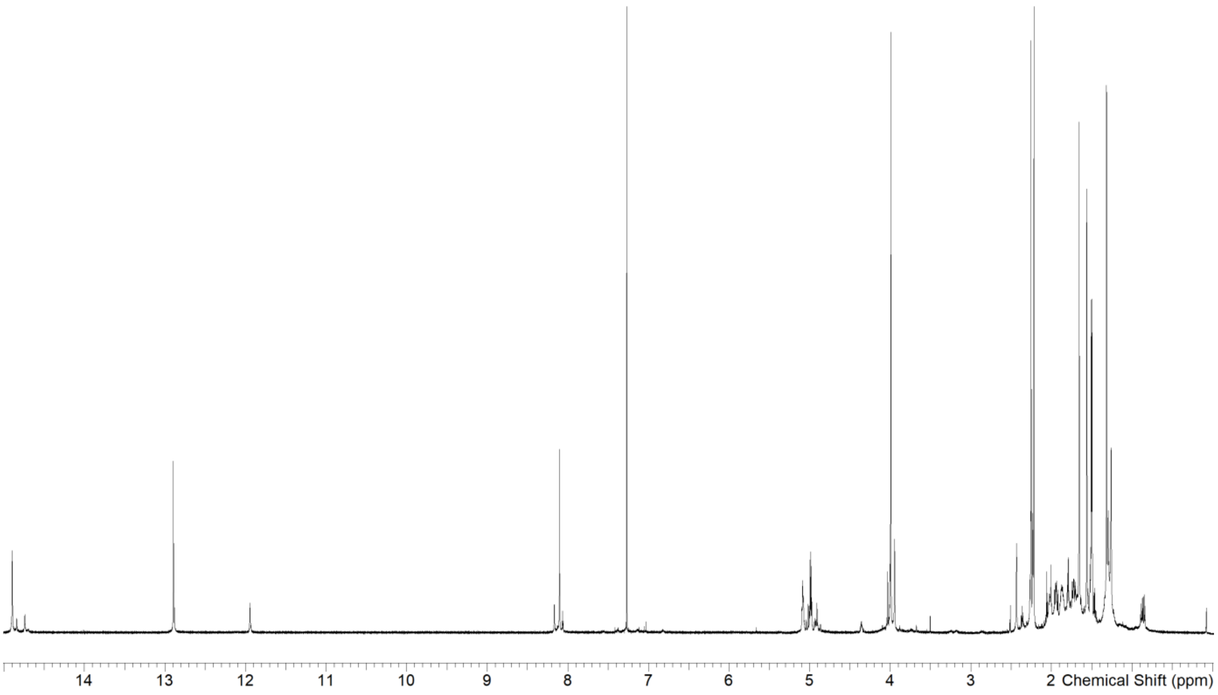

Figure S9: ^1H-NMR spectrum (500 MHz, $CDCl_3$) of furaquinocin L.

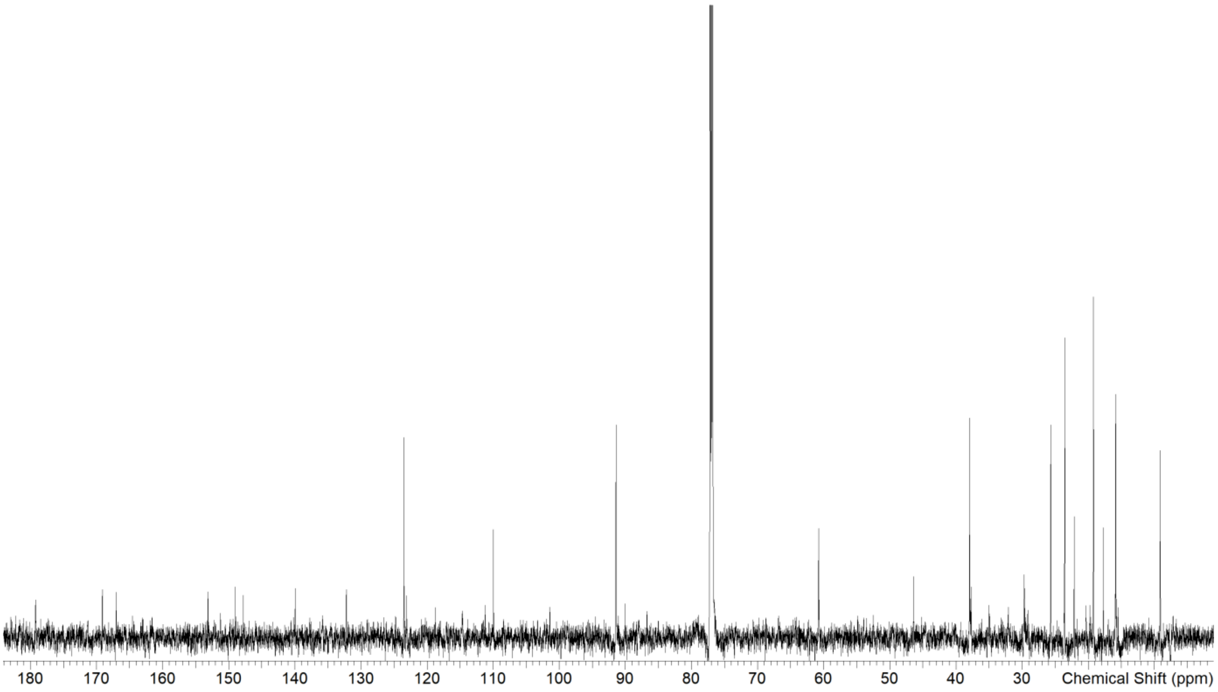

Figure S10: ^{13}C-NMR spectrum (125 MHz, $CDCl_3$) of furaquinocin L.

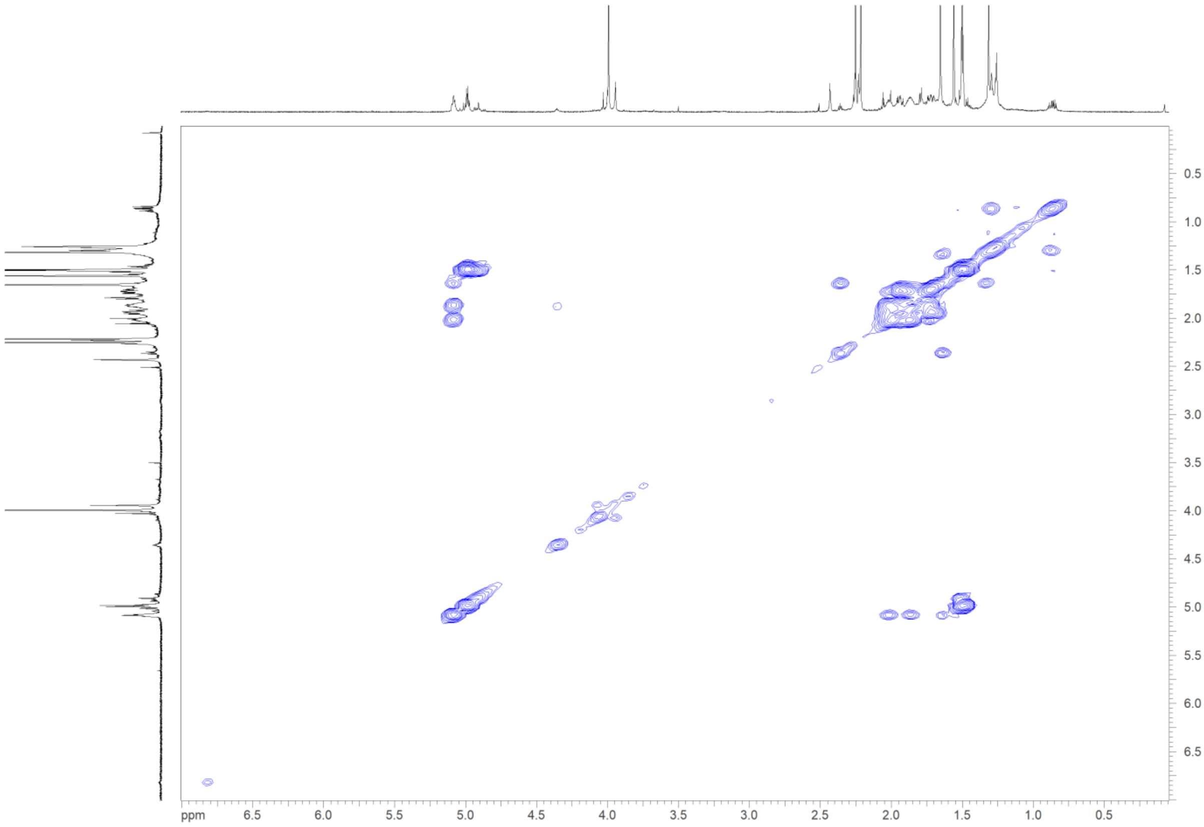

Figure S11: ^{1}H-^{1}H-COSY spectrum ($CDCl_3$) of furaquinocin L.

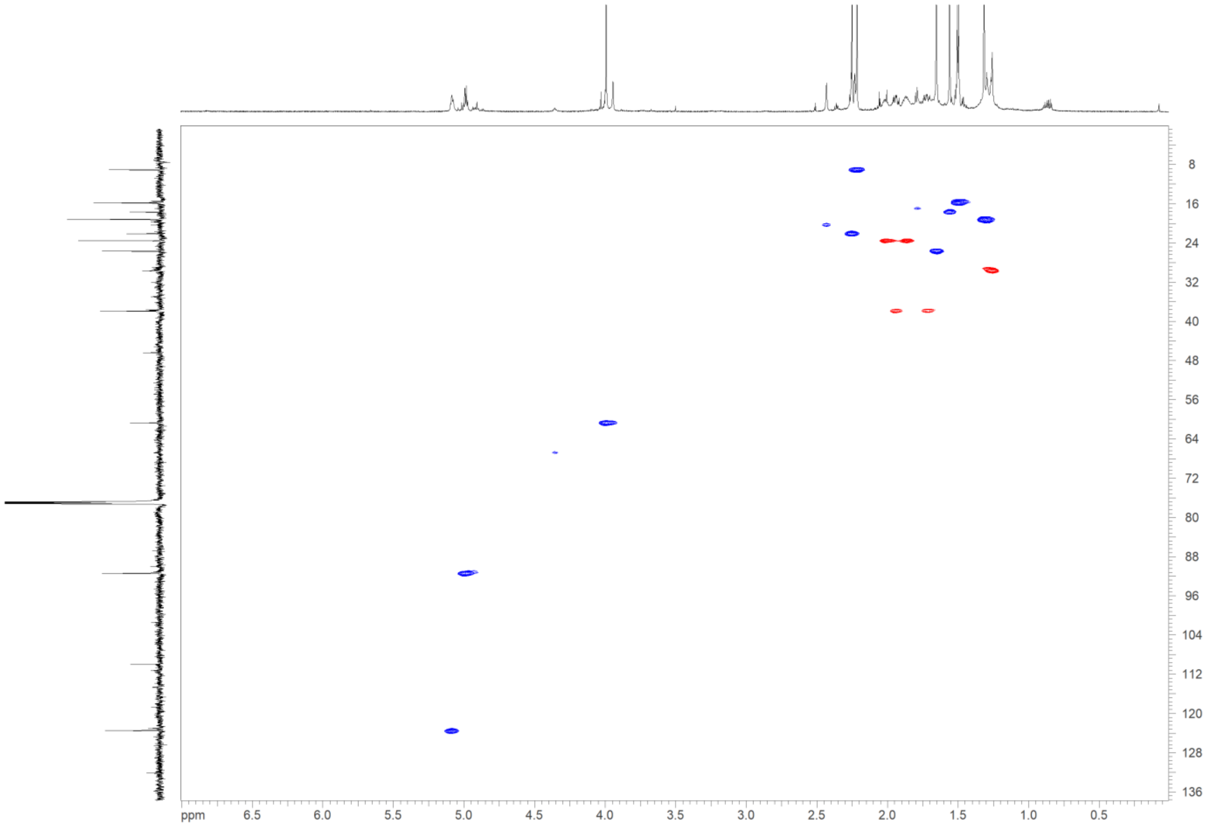

Figure S1: Edited HSQC spectrum ($CDCl_3$) of furaquinocin L.

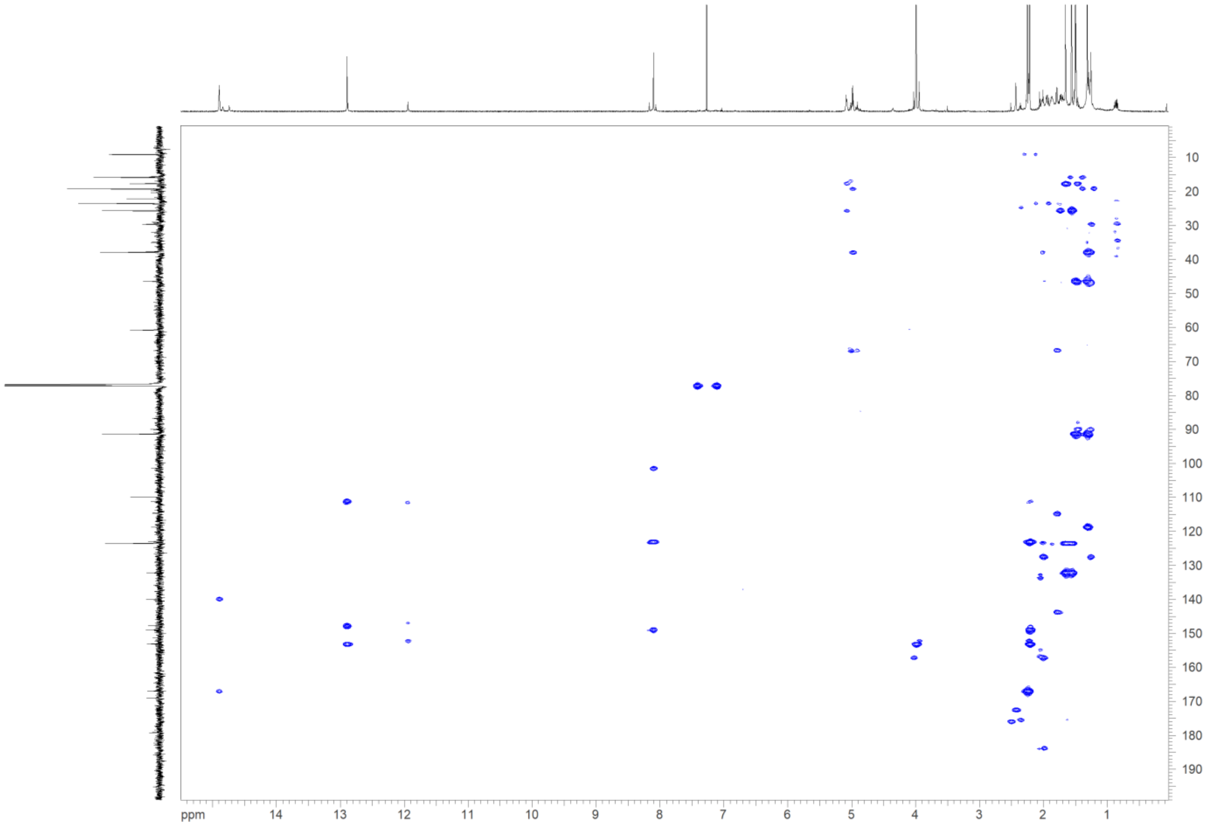

Figure S13: $^{1}H,^{13}C$-HMBC spectrum ($CDCl_3$) of furaquinocin L.

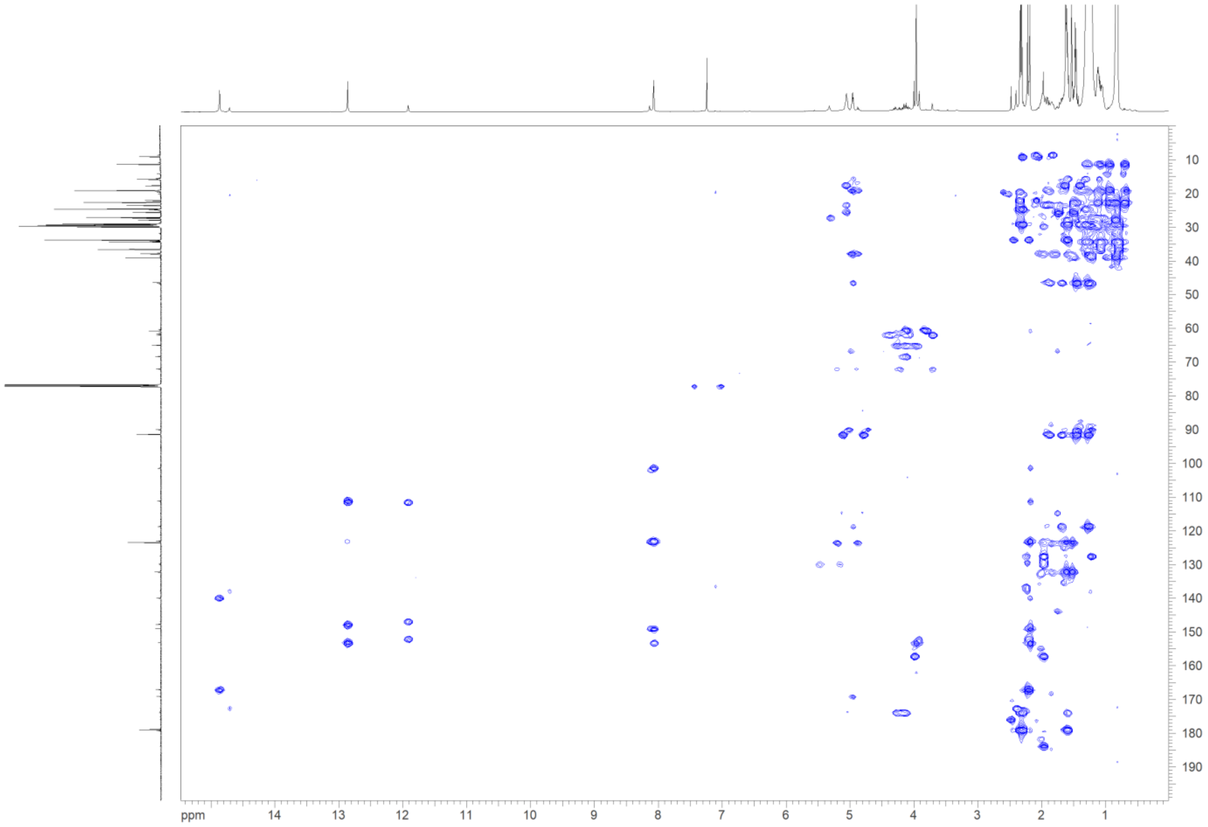

Figure S14: HMBC spectrum ($CDCl_3$) of furaquinocin L with impurities but higher quantity.

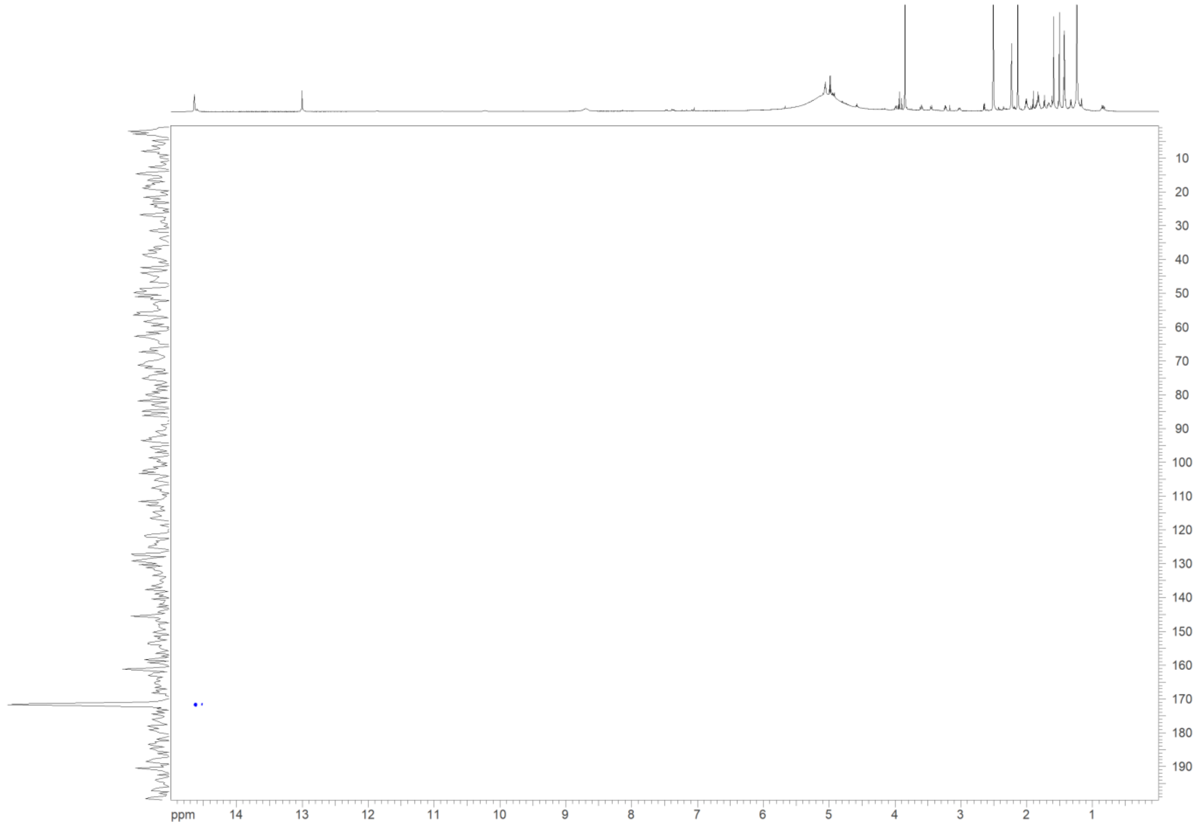

Figure S15: $^{1}H,^{15}N$-HSQC spectrum (DMSO-d_6, TFA) of furaquinocin L.

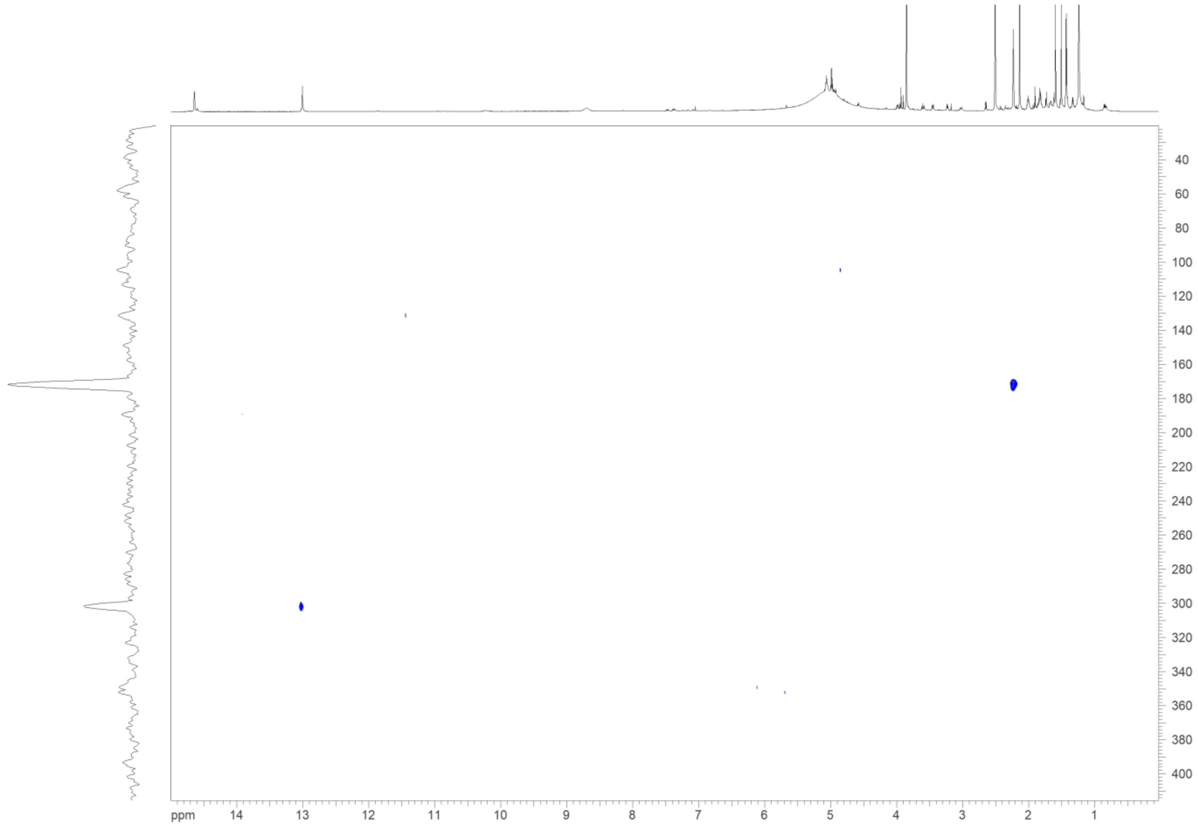

Figure S16: $^{1}H,^{15}N$-HMBC spectrum (DMSO-d_6, TFA) of furaquinocin L.

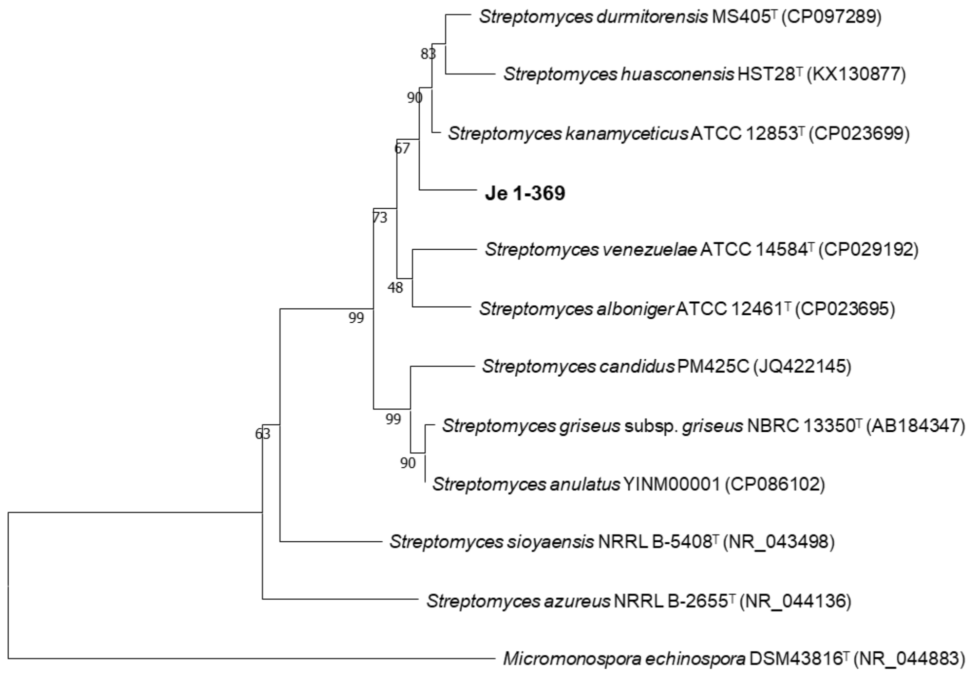

Figure S17: NJ tree based on the 16S rRNA gene sequences of the Je1–369 (in bold), its closest neighbors and selected Streptomyces-type strains. *Micromonospora echinospora* DSM43816 was used as an outgroup.

Table S1: Secondary metabolite biosynthesis gene clusters identified in the genome of *Streptomyces* sp. Je 1-369

Region	Type	Most similar known cluster/MIBiG BGC-ID	Similarity (%)
Region 1	T2PKS	Fluostatin/ BGC0001904	74
Region 2	T3PKS, other, phenazine, terpene	Furaquinocin/ BGC0001078	60
Region 3	T1PKS	Oxalomycin B/ BGC0001106	6
Region 4	RiPP-like		
Region 5	T3PKS	Violapyrone B/ BGC0001905	28
Region 6	NRPS,T1PKS	Acyldepsipeptide/ BGC0001967	15
Region 7	Ectoine	Netropsin/ BGC0000327	13
Region 8	NRPS	Mirubactin/ BGC0000392	50
Region 9	Terpene	Isorenieratene/ BGC0001456	100
Region 10	Ectoine	Ectoine/ BGC0000853	100
Region 11	NRPS,T1PKS	Platencin/ BGC0001156	6
Region 12	Lanthipeptide class III	Informatipeptin/ BGC0000518	42
Region 13	Betalactone	7-deoxypactamycin/ BGC0000119	5
Region 14	Melanin	Melanin/ BGC0000910	100
Region 15	NRPS, T1PKS	Vazabitide/ BGC0001818	30
Region 16	PKS-like, amglyccycl, butyrolactone	Cetoniacytone A/ BGC0000283	12
Region 17	Terpene	Albaflavenone/ BGC0000660	100
Region 18	T1PKS	Stambomycin A/ BGC0000151	20
Region 19	Terpene	Ribostamycin/ BGC0000713	7
Region 20	Siderophore		
Region 21	NRPS	Coelichelin/ BGC0000325	100
Region 22	Terpene	2-methylisoborneol/BGC0000657	75
Region 23	RiPP-like		
Region 24	Terpene	Geosmin/ BGC0001181	100
Region 25	NRPS, NRPS-like	Cadaside/ BGC0001968	23
Region 26	T3PKS, other, phenazine	Endophenazine/ BGC0001080	77
Region 27	Terpene	Kanamycin/ BGC0000703	6
Region 28	Terpene	Hopene/ BGC0000663	84
Region 29	T1PKS	Monensin/ BGC0001670	100
Region 30	Other	A-503083 A/ BGC0000288	7
Region 31	RiPP-like		
Region 32	NRPS, T3PKS	Feglymycin/ BGC0001233	78
Region 33	Terpene	Merochlorin/ BGC0001083	7
Region 34	T1PKS, hglE-KS, terpene	Rifamorpholine/ BGC0001759	9
Region 35	T3PKS, other, phenazine, terpene	Furaquinocin/ BGC0001078	60
Region 36	T2PKS	Fluostatin/ BGC0001904	74
